# Increased macrolide resistance rate of *Mycoplasma pneumoniae* correlated with epidemic in Beijing, China in 2023

**DOI:** 10.3389/fmicb.2024.1449511

**Published:** 2024-08-06

**Authors:** Yujie Chen, Xinyu Jia, Yagang Gao, Xue Ren, Bing Du, Hanqing Zhao, Yanling Feng, Guanhua Xue, Jinghua Cui, Lin Gan, Junxia Feng, Zheng Fan, Tongtong Fu, Ziying Xu, Zihui Yu, Yang Yang, Shuo Zhao, Lijuan Huang, Yuehua Ke, Ling Cao, Chao Yan, Jing Yuan

**Affiliations:** ^1^Department of Bacteriology, Capital Institute of Pediatrics, Beijing, China; ^2^Graduate School of Peking Union Medical College, Beijing, China

**Keywords:** *Mycoplasma pneumoniae*, genotypes, macrolide-resistance, epidemic, children

## Abstract

We collected respiratory specimens from 128 pediatric patients diagnosed with pneumonia in Beijing in late 2023. *Mycoplasma pneumoniae* was detected in 77.3% (99/128) patients, with 36.4% (4/11), 82.9% (34/41), 80.3% (61/76) in children aged less than 3 years, 3–6 years, over 7 years, respectively. *Mycoplasma pneumoniae* (*M. pneumoniae*) was characterized using P1 gene typing, MLVA typing and sequencing of domain V of the 23S rRNA gene. P1 gene type 1 (P1-1; 76.1%, 54/71) and MLVA type 4-5-7-2 (73.7%, 73/99) were predominant. MLVA identified a new genotype: 3–4–6-2. Macrolide resistance-associated mutations were detected in 100% of samples, with A2063G accounting for 99% and A2064G for 1%. The positive rate of *M. pneumoniae* was higher compared to previous reports, especially in children less than 3 years, suggesting a *M. pneumoniae* epidemic showing a younger age trend occurred in late 2023 in Beijing, China. Higher proportions of macrolide-resistant *M. pneumoniae*, P1-1 and 4-5-7-2 genotype *M. pneumoniae* indicated increased macrolide resistance rate and genotyping shift phenomenon, which might be attributable to this epidemic. Additionally, complete clinical information from 73 *M. pneumoniae* pneumonia inpatients were analyzed. The incidence of severe *M. pneumoniae* pneumonia was 56.2% (41/73). *Mycoplasma pneumoniae* pneumonia patients exhibited longer duration of fever, with a median value of 10.0 days (IQR, 8.0–13.0), and higher incidence of complications (74.0%, 54/73). However, in this cohort, we found that the severity of *M. pneumoniae* pneumonia, co-infection, or complications were not associated with *M. pneumoniae* P1 gene or MLVA types. Clinicians should be aware that patients infected with macrolide-resistant *M. pneumoniae* exhibited more severe clinical presentations.

## 1 Introduction

*Mycoplasma pneumoniae* or *Mycoplasmoides pneumoniae* (*M. pneumoniae*), an important pathogen causing human upper and lower respiratory tract infections, is the leading cause of community-acquired pneumonia in school-age children and adolescents, accounting for 10%–40% of cases (Waites et al., 2017; Gupta et al., 2018; Wang et al., 2022). *Mycoplasma pneumoniae* pneumonia (MPP) is typically mild and sometimes self-limiting, however, the incidence of severe MPP (SMPP) requiring hospitalization has increased in recent years (Izumikawa, 2016; Yan et al., 2019a; Conroy, 2023).

Globally, *M. pneumoniae* epidemics exhibit an obvious periodic pattern, emerging every 3–7 years, with each outbreak lasting 1–2 years (Sun et al., 2013; Beeton et al., 2020; Yan et al., 2024). To monitor such epidemics and identify strain diversity within outbreaks at the molecular level, P1 gene typing using polymerase chain reaction restriction fragment length polymorphism analysis (PCR-RFLP), multiple-locus variable-number tandem-repeat analysis (MLVA), and sequencing of domain V of the 23S rRNA gene are the most widely used methods (Yan et al., 2019b). Based on differences in the P1 adhesin gene, *M. pneumoniae* strains has been classified into type 1 (P1-1) and type 2 (P1-2), and other variants. MLVA has since been developed and amended, offering higher discriminatory power than P1 gene typing. The proportion of each subtype has fluctuated over time and with the epidemic area (Zhao et al., 2011; Sun et al., 2013; Kenri et al., 2023).

For many years, macrolides were used as first-line antibiotics for treating *M. pneumoniae* infections in children (Waites et al., 2017). Because of their wide usage, macrolide-resistant *M. pneumoniae* (MRMP) emerged and has spread globally for more than 20 years, with prevalence rates ranging from less than 30% in Europe and the United States, to 24.6–50.1% in Japan, and over 90% in China (Okazaki et al., 2001; Ando et al., 2018; Katsukawa et al., 2019; Xiao et al., 2020; Loconsole et al., 2021; Wang et al., 2022; Kenri et al., 2023; Xu et al., 2024). Mutations at position 2063 and 2064 within domain V of the 23S rRNA gene are mainly responsible for macrolide resistance (Yan et al., 2019a; Wang et al., 2022). MRMP infections lead to longer duration of fever and hospitalization compared with macrolide-sensitive *M. pneumoniae* infections, and have increased the proportion of patients changed to treatment with second-line antibiotics, such as fluoroquinolones and tetracyclines (Pereyre et al., 2016).

From April to September 2023, the incidence of *M. pneumoniae* infection increased in many countries, especially in Asia and Europe (Meyer Sauteur et al., 2023). In this study, we aimed to test the rate of *M. pneumoniae*-positivity among pediatric pneumonia patients in Beijing in late 2023, analyze the molecular characteristics of *M. pneumoniae* and clinical features of MPP.

## 2 Materials and methods

### 2.1 Specimen collection

Respiratory specimens, including sputum, nasopharyngeal swabs, and bronchoalveolar lavage fluid, were collected from pediatric patients diagnosed with pneumonia from October to November of 2023 in the Affiliated Children’s Hospital of Capital Institute of Pediatrics in Beijing. This study was approved by the research board of the Ethics Committee of the Capital Institute of Pediatrics in Beijing and informed consent was obtained for the collection of all specimens.

### 2.2 Specimen DNA and RNA extraction

DNA was extracted from 400 μL of each sample using TIANamp Bacteria DNA Kit (Tiangen Biotech Co., Ltd. Beijing, China), in accordance with the manufacturer’s instructions. DNA extracted from the reference strain M129 (ATCC 29342) and FH (ATCC 15531) were used as positive controls. Double distilled water was used as a negative control. DNA was immediately used or stored at −20°C. RNA was extracted using QIAamp® Viral RNA Mini Kit (Qiagen, Hilden, Germany) according to the manufacturer’s instructions. RNA was immediately used or stored at −80°C.

### 2.3 Detection of *Mycoplasma pneumoniae* and macrolide resistance-associated mutations

*Mycoplasma pneumoniae* and the macrolide resistance-associated mutations A2063G or A2064G were detected in clinical specimens using *Mycoplasma pneumoniae* and Macrolide-Resistant isolates Diagnostic Kit (PCR Fluorescence Probing; Mole BioScience Co., Ltd. Jiangsu, China), in accordance with the manufacturer’s instructions. To distinguish between mutations at sites 2063 and 2064, and to detect mutations at other sites (2067, 2611, 2616, and 2617), domain V of the 23S rRNA gene was PCR-amplified and sequenced, as previously described (Lin et al., 2010).

### 2.4 P1 gene and MLVA typing

P1 gene typing was performed using nested PCR and RFLP analysis with *Hae*III digestion, as previously described (Sun et al., 2013). Products of type 2 specimens were sequenced to identify type 2 variants. Variable-number tandem-repeat loci Mpn13, Mpn14, Mpn15, and Mpn16 were amplified and sequenced, with the results displayed in the form of n-n-n-n, in which ‘n’ represents the repeat number at each locus (Dumke and Jacobs, 2011).

### 2.5 Detection of co-infection

*Chlamydia pneumoniae* and respiratory virus including *Influenza virus*, *Respiratory syncytial virus*, *Human parainfluenza virus*, *Adenovirus*, *Human bocavirus*, *Human metapneumovirus*, *Human coronavirus* and *Rhinovirus* were examined using Resp®13 Respiratory Pathogen Multiplex Kit (Ningbo Health Gene Technologies Co., Ltd. Zhejiang, China), in accordance with the manufacturer’s instructions. *Epstein–Barr virus*, and *Cytomegalovirus* were examined using *Epstein–Barr virus* Nucleic Acid Quantitative Detection Kit and *Cytomegalovirus* Nucleic Acid Quantitative Detection Kit (Daan Gene Co., Ltd. Guangzhou, China). Bacteria including *Streptococcus pneumoniae, Staphylococcus aureus, Methicillin resistant Staphylococcus, Klebsiella pneumoniae, Haemophilus influenzae, Pseudomonas aeruginosa, Acinetobacter baumannii* and *Stenotrophomonas maltophilia* were identified using the Respiratory Pathogens Nucleic Acid Detection Kit (CapitalBio Technology Co., Ltd. Beijing, China), in accordance with the manufacturers’ protocols. Fungi were cultured by inoculating specimens on Sabouraud’s agar medium for 48 h at 37°C with 5% CO_2_.

### 2.6 Clinical information

For *M. pneumoniae*-positive specimens, we analyzed the corresponding patient information, including age, sex, clinical symptoms, laboratory detection, radiological findings, duration of fever, cough and hospital stay. SMPP was diagnosed based on fever (>39.0°C), dyspnea, oxygen saturation, radiological deterioration or consolidation present in ≥2/3 of the lung lobes, and extrapulmonary complications, according to the “Guidelines for Diagnosis and Treatment of *Mycoplasma pneumoniae* Pneumonia in Children (2023 edition)” designated by National Health Commission of China (2023). Other cases were diagnosed as general *M. pneumoniae* pneumonia (GMPP).

### 2.7 Statistical analysis

SPSS 26.0 software (IBM, New York, United States) was used for statistical analysis. The distribution of variables was evaluated by the Shapiro–Wilk test. Continuous data were described using medians and interquartile ranges (IQRs). Continuous variables were compared using independent-sample t tests or Mann–Whitney U tests. To compare categorical variables, we used descriptive statistics, including the Chi-squared test and Fisher exact test. *p* value <0.05 was considered to indicate statistical significance.

## 3 Results

### 3.1 *Mycoplasma pneumoniae* detection and general information analysis

In total, we collected 128 respiratory samples: 82.0% (105/128) from bronchoalveolar lavage fluid, 15.6% (20/128) from sputum and 2.34% (3/128) from nasopharyngeal swabs. Each sample corresponded to one patient. Of all patients, (70.3%) 90/128 were inpatients and 29.7% (38/128) were outpatients. *Mycoplasma pneumoniae* was detected in 77.3% (99/128) of samples, in which 73.7% (73/99) were from inpatients and 26.3% (26/99) were from outpatients. The median age of *M. pneumoniae-*positive patients was 7.5 years old (IQR, 6.5–10.0), of whom 52.5% (52/99) were male and 47.5% (47/99) were female (male: female ratio = 1.1). The *M. pneumoniae* detection rate was significantly higher in children above 3 years of age, with the highest positive rate in the 3–6-year age group. There was no significant difference in *M. pneumoniae* positivity between the sexes (Table 1).

**Table 1:** Detection rate of *Mycoplasma pneumoniae* and A2063G/A2064G in different age and sex groups.

	No. of patients	No. of *M. pneumoniae* positive (%)	No. of A2063/2064G positive (%)
Male	63	52(82.5%)	52(100%)
Female	65	47(72.3%)	47(100%)
<3 years	11	4(36.4%)^*^	4(100%)
3–6 years	41	34(82.9%)	34(100%)
>7 years	76	61(80.3%)	61(100%)
Total	128	99(77.3%)	99(100%)

*Mycoplasma pneumoniae* positive rate in different age groups: *p* = 0.008. ^*^Positive rate of *M. pneumoniae* in < 3 years old was significantly lower than other groups. *Mycoplasma pneumoniae* positive rate in different gender groups: *p* = 0.167.

### 3.2 Molecular characteristics of *Mycoplasma pneumoniae*

#### 3.2.1 P1 gene typing

**Figure 1:**
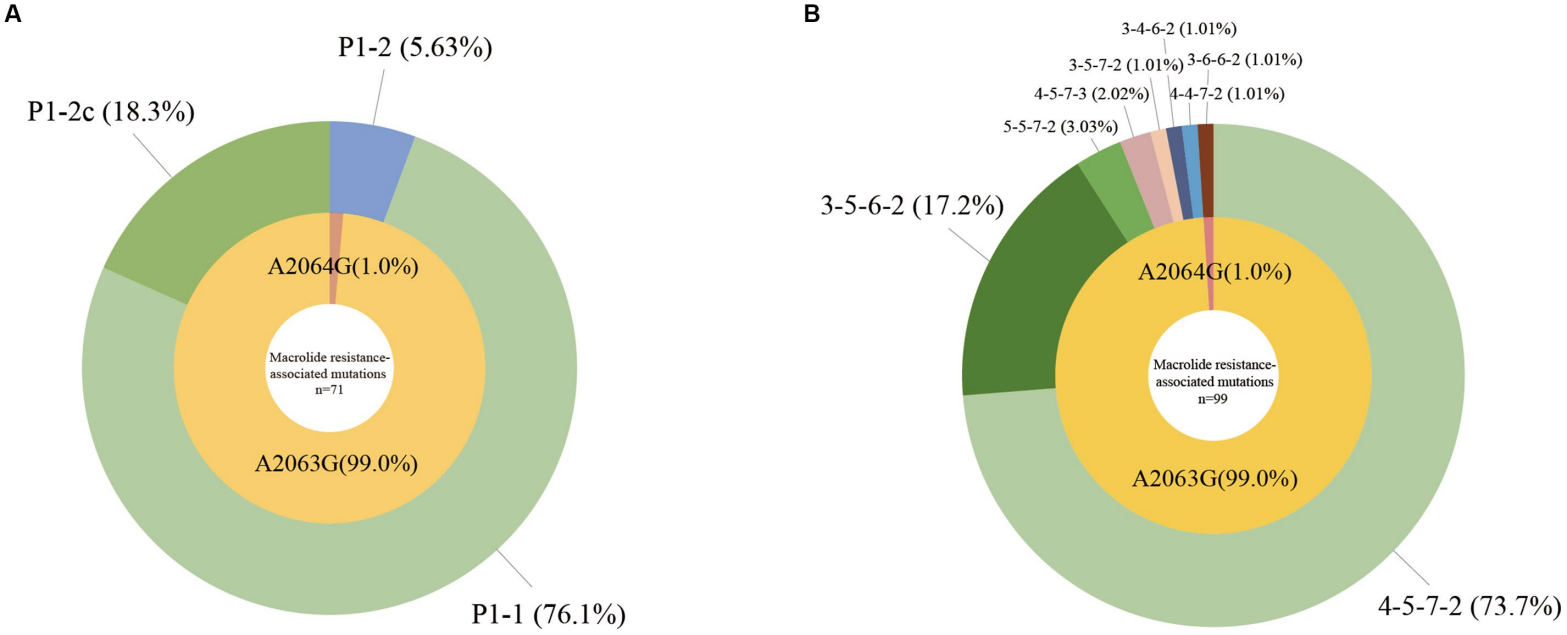
Molecular characteristics of *Mycoplasma pneumoniae*. **(A)** P1 gene types and macrolide resistance-associated mutations of *M. pneumoniae*, *n* = 71. **(B)** MLVA types and macrolide resistance-associated mutations of *M. pneumoniae*, *n* = 99.

Among 99 *M. pneumoniae*-positive samples, we successfully identified the P1 gene types in 71 samples. P1-1 was the predominant subtype, accounting for 76.1% (54/71) of cases, with the remaining 18.3% (13/71) classified as P1-2c, 5.63% (4/71) classified as P1-2 (Figure 1A; Table 2).

**Table 2:** Molecular profiles of 99 *Mycoplasma pneumoniae* samples collected from October and November in 2023 in Beijing, China.

	4-5-7-2	3-5-6-2	5-5-7-2	4-5-7-3	3-5-7-2	3-6-6-2	4-4-7-2	3-4-6-2	Total
P1-1	49(A2063G)	-	3(A2063G)	2(A2063G)	-	-	-	-	54
P1-2	1(A2063G)	2(A2063G)	-	-	-	1(A2064G)	-	-	4
P1-2c	-	12(A2063G)	-	-	1(A2063G)	-	-	-	13
P1 gene typing failed	23(A2063G)	3(A2063G)	-	-	-	-	1(A2063G)	1(A2063G)	28
Total	73	17	3	2	1	1	1	1	99

#### 3.2.2 MLVA typing

We detected eight MLVA types among the 99 *M. pneumoniae*-positive samples, 73.7% (73/99) were 4–5–7-2, 17.2% (17/99) were 3–5–6-2, 3.03% (3/99) were 5–5–7-2, 2.02% (2/99) were 4–5–7-3, with 1.01% (1/99) each of 3–5–7-2, 3–6–6-2, and 4–4–7-2. Particularly, we detected a novel type 3–4–6-2, which accounted for 1.01% (1/99) in the *M. pneumoniae*-positive samples (Figure 1B; Table 2).

#### 3.2.3 Macrolide resistance-associated mutations

Using multiple fluorescence PCR, A2063G or A2064G was detected in 100% (99/99) *M. pneumoniae*-positive samples. Through nested PCR and sequencing, all 99 samples were confirmed to harbor either the A2063G (99.0%, 98/99) or the A2064G (1.0%, 1/99) mutation. Mutations at other sites were not detected in this study (Figures 1A,B; Table 2).

### 3.3 Clinical features of *Mycoplasma pneumoniae*-positive patients

#### 3.3.1 Clinical symptoms and laboratory results

**Figure 2:**
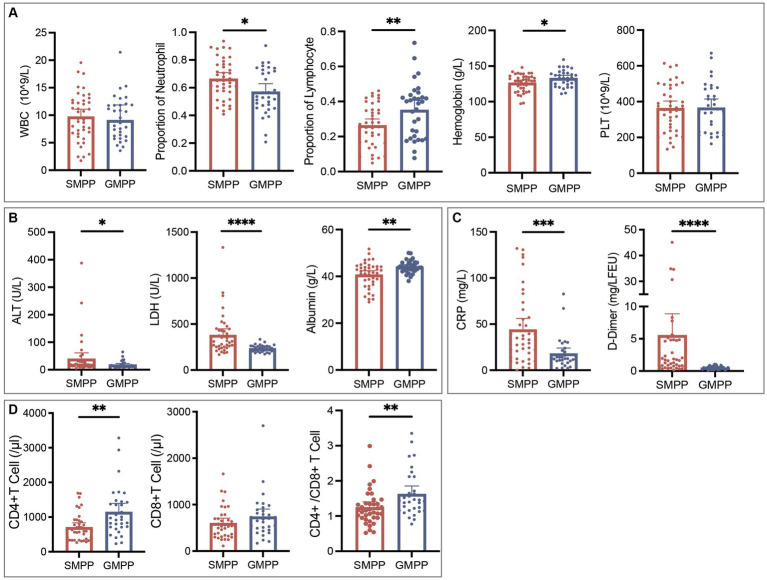
Comparisons of laboratory results of SMPP and GMPP patients. **(A)** Blood routine examination results. **(B)** Alanine aminotransferase (ALT), lactate dehydrogenase (LDH) and albumin results. **(C)** C-reactive protein (CRP) and D-Dimer results. **(D)** Immune cell counts. ^*^p < 0.05, ^**^p < 0.01, ^***^p < 0.001, ^****^p < 0.0001.

In total, we collected complete medical records from 73 *M. pneumoniae*-positive inpatients, of whom 56.2% (41/73) were diagnosed with SMPP and 43.8% (32/73) with GMPP. As shown in Table 3, no age or sex differences were found between the SMPP and GMPP groups. All patients had fever and cough symptoms. Wheezing, chest pain, vomiting and disturbance of consciousness were relatively rare, and were mainly observed in SMPP patients. For all patients, the median values for duration of fever, duration of cough, and the length of hospital stay were 10.0 (IQR, 8.0–13.0), 12.0 (15.0–18.0) and 4.0 (3.0–7.0) days, respectively, and were significantly longer in SMPP patients. Additionally, compared with GMPP patients, SMPP patients had significantly higher levels of neutrophil rate, C-reactive protein (CRP), lactate dehydrogenase (LDH), alanine aminotransferase (ALT) and D-Dimer, but significantly lower lymphocyte and hemoglobin levels, CD4(+) T cell counts, and CD4/CD8 ratios. No significant differences were found in white blood cell or platelet counts (Figure 2; Supplementary Table 1). We analyzed the associations between the severity of MPP and genotypes of *M. pneumoniae,* but no significances were found (Supplementary Table 2).

**Table 3:** Clinical information of SMPP and GMPP.

	Total (*n* = 73)	SMPP (*n* = 41)	GMPP (*n* = 32)	*P-*value
Age	7.5 (6.5–10.2)	7.3 (6.1–10.1)	8.7 (6.6–10.8)	0.245
Sex ratio (male/female)	0.9 (35/38)	1.1 (21/20)	0.8 (14/18)	0.526
**Clinical presentation**				
Fever	100% (73/73)	100% (41/41)	100% (32/32)	
Cough	100% (73/73)	100% (41/41)	100% (32/32)	
Wheezing	5.48% (4/73)	9.76% (4/41)	-	
Chest pain	2.74% (2/73)	4.88% (2/41)	-	
Vomiting	2.74% (2/73)	2.44% (1/41)	3.13% (1/32)	
Disturbance of consciousness	1.37% (1/73)	2.44% (1/41)	-	
Co-infection	41.1% (30/73)	48.8% (20/41)	31.3% (10/32)	0.131
Length of stay	4.0 (3.0–7.0)	6.0 (4.0–9.0)	3.0 (3.0–4.0)	0.000
Fever duration	10.0 (8.0–13.0)	12.0 (9.0–15.0)	8.0 (6.0–9.8)	0.000
Cough duration	12.0 (15.0–18.0)	17.0 (14.0–19.8)	13.0 (11.3–17.8)	0.018

#### 3.3.2 Associations between co-infection and *Mycoplasma pneumoniae* molecular characteristics

**Figure 3:**
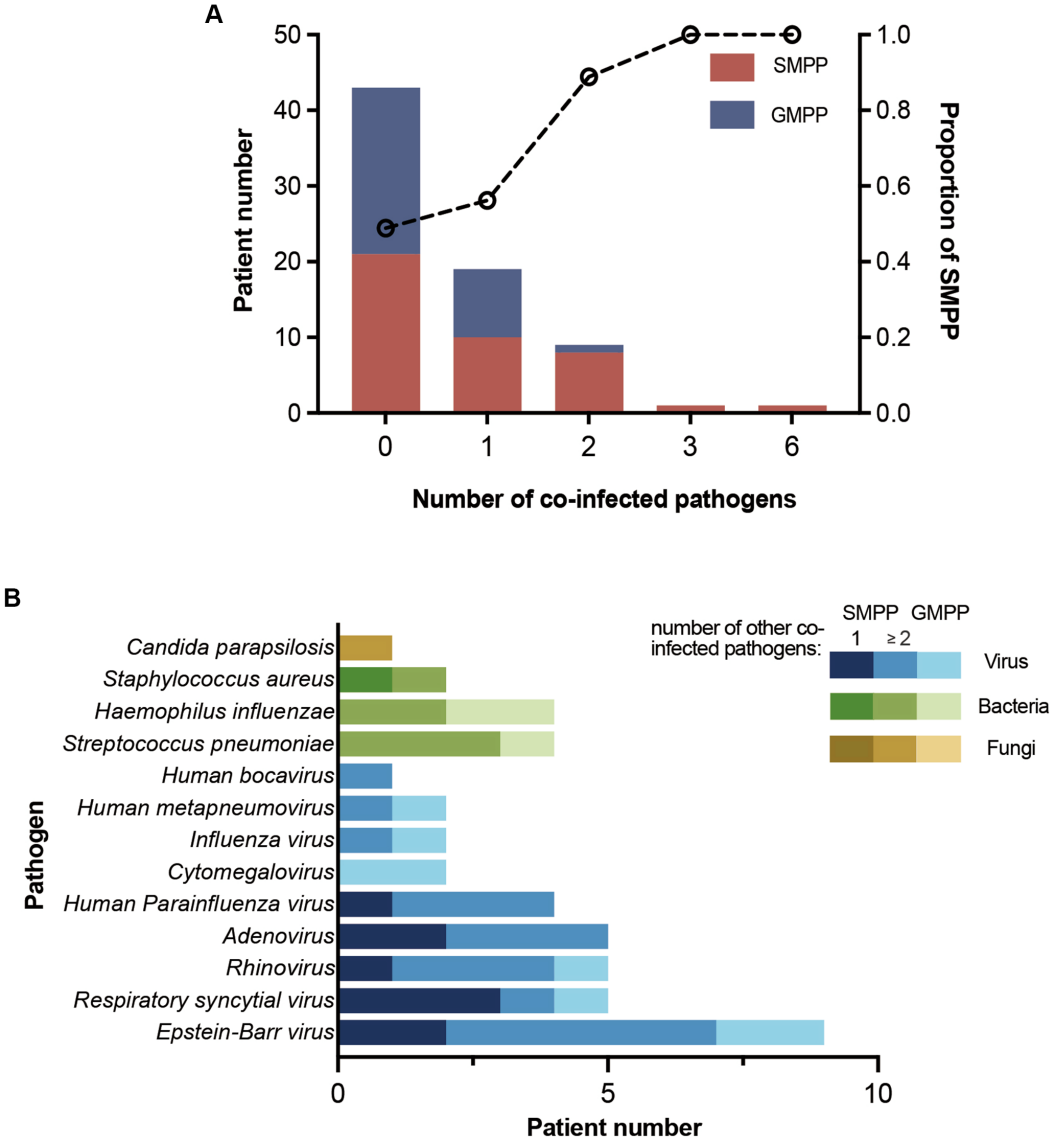
Patient number of co-infection and co-infected pathogens. **(A)** Number of SMPP and GMPP patients with different numbers of co-infected pathogens. The proportion of SMPP patients increased with the number of co-infected pathogens. **(B)** Number of patients co-infected with different pathogens. Patients are divided into SMPP and GMPP patients. SMPP patients are divided into patients co-infected with one other pathogen and ≥2 other pathogens.

Overall, 41.1% (30/73) *M. pneumoniae-*positive inpatients were co-infected with other pathogens. Patients with co-infection were significantly younger than those with *M. pneumoniae*-mono infection (Supplementary Table 3). Most co-infected patients were co-infected with one other pathogen, and the proportion of SMPP disease increased with the number of co-infected pathogens (Figure 3A). In total, 13 pathogens were detected in all patients with co-infection: nine viruses, three bacteria, and one fungus. Among them, *Epstein–Barr virus* (30.0%, 9/30) was the predominant co-infected pathogen. *Respiratory syncytial virus* was the most common detected pathogen in SMPP patients co-infected with one other pathogen, accounting for 30.0% (3/10) cases (Figure 3B). No statistically significant associations were found between *M. pneumoniae* genotypes and co-infection (Supplementary Table 3).

#### 3.3.3 Associations between complications and *Mycoplasma pneumoniae* molecular characteristics

Of all *M. pneumoniae-*positive inpatients, 74.0% (54/73) had complications. The predominant extrapulmonary and intrapulmonary complications were blood system diseases (61.6%, 45/73) and pulmonary consolidation (53.4%, 39/73). SMPP patients accounted for higher proportions of patients with these two complications (Table 4). Other extrapulmonary complications included liver dysfunction (15.1%, 11/73), myocardial or pericardial damage (13.7%, 10/73), hypoxemia (13.7%, 10/73), digestive disorder (9.59%, 7/73), electrolyte disturbance (8.22%, 6/73), skin rash (4.11%, 3/73), splenomegaly (2.74%, 2/73), vasculitis (1.37%, 1/73), encephalitis (1.37%, 1/73), thyroid dysfunction (1.37%, 1/73), and acute kidney injury (1.37%, 1/73). Other intrapulmonary complications included pleural effusion (19.2%, 14/73), atelectasis (11.0%, 8/73), plastic bronchitis (6.85%, 5/73) and necrosis (4.11%, 3/73). Excluding patients with co-infection or congenital diseases, we looked for associations between genotypes of *M. pneumoniae* and the predominant complications but found none (Supplementary Table 4).

**Table 4:** Number of SMPP and GMPP patients with blood system diseases and pulmonary consolidation.

	SMPP patient	GMPP patient	Total No. of patient
Blood system diseases	71.1% (32/45)	28.9% (13/45)	45
Anemia	78.6% (11/14)	21.4% (3/14)	14
Abnormal coagulation function	81.8% (30/37)	18.2% (7/37)	37
Leukopenia	100% (5/5)	-	5
Thrombocytosis	25.0% (1/4)	75.0% (3/4)	4
Pulmonary consolidation	53.8% (21/39)	46.2% (18/39)	39

#### 3.3.4 Anti-*Mycoplasma pneumoniae* therapy

Excluding patients with co-infection, congenital diseases, or drug allergy, we analyzed the drug treatment of the remaining 41 patients with the aim of looking into the efficacy of macrolides against *M. pneumoniae* in this epidemic. Forty patients were treated first with azithromycin; only one patient was treated first with minocycline. However, 100% (40/40) of the azithromycin-treated patients were still febrile without significant improvements at 72 h post-azithromycin administration. Furthermore, 75.0% (30/40) of these patients were finally switched to other classes of antibiotic drugs including levofloxacin, minocycline and doxycycline which were effective in improving body temperature.

## 4 Discussion

*Mycoplasma pneumoniae*, a common pathogen of respiratory infection in children, causes epidemics with a recognized cycle interval of 3–7 years. In Beijing, the detection rate of *M. pneumoniae* in children in non-epidemic periods has ranged from 11.69%–21.1%, and increased to 47.1%–56.8% in epidemic periods from 2010 to 2018 (Zhao et al., 2014; Yan et al., 2016, 2019b; Cheng et al., 2022). During the most recent worldwide COVID-19 pandemic, which occurred in late 2019 and 2020, the prevalence of *M. pneumoniae* infection decreased significantly. However, since April 2023, the incidence of *M. pneumoniae* infection has increased and steep rises of *M. pneumoniae* infection occurred in China, Denmark, and the Netherlands in October 2023 (Meyer Sauteur et al., 2023; Bolluyt et al., 2024; Gong et al., 2024; Nordholm et al., 2024). In October 2023 in Beijing, there was a sharp increase in the number of pediatric patients diagnosed with acute respiratory tract infections and pneumonia, with the detection rate of *M. pneumoniae* exceeding 61.1% among inpatients (Gong et al., 2024; Yan et al., 2024). In our study, 77.3% (99/128) specimens were positive for *M. pneumoniae* infection, suggesting an outbreak occurred in Beijing in late 2023. It is clearly higher than previously reported data.

The incidence of *M. pneumoniae* infection varies in different pediatric age groups, generally showing higher prevalence in children over 5 years old of age, and lower prevalence in those under 3 years of age (Zhao et al., 2014; Kutty et al., 2019). In previous studies, detection rates in Beijing peaked in the 7–13-year age group from 2007 to 2012, and in the 10–14-year age group from 2015 to 2020 (Zhao et al., 2014; Wang et al., 2022). During the epidemic of 2023, children aged 5–11 years and 6–12 years had the highest detection rates in the Netherlands and Denmark, respectively (Bolluyt et al., 2024; Nordholm et al., 2024). In our study, the detection rate in children under 3 years of age was significantly lower than that in other pediatric age groups, with the highest detection rate in children aged 3–6 years. Although the detection rate in children under 3 years of age in this study was significantly lower than that in other pediatric age groups, it was higher than that in previous studies, suggesting a trend in younger-age *M. pneumoniae* infection in 2023 in Beijing, which is consistent with our previous report (Zhao et al., 2014; Yan et al., 2019a; Cheng et al., 2022; Yan et al., 2024).

With the widespread incidence of MRMP, single-nucleotide mutations in the domain V region of the 23S rRNA gene have been monitored worldwide. In the United States, the incidence of MRMP was 8.3% from 2012 to 2018, in which 86.5% harbored the A2063G mutation, and 7.1% from 2023 to 2024 (Xiao et al., 2020; Edens et al., 2024). From 2016 to 2020 in Switzerland, a study including 54 participants found that only one patient was infected with MRMP carrying the A2063G mutation (Meyer Sauteur et al., 2021). From 2019 to 2020 in Japan, mutations in the 23S rRNA gene were detected in 24.6% isolates, of which 89.7%, 6.9%, and 3.4% harbored the A2063G, A2064G, and A2063T mutations (Kenri et al., 2023). In different regions of China, the incidence of MRMP has varied from 84.72 to 97.1%, with the A2063G mutation accounting for more than 99% of cases (Wang et al., 2022; Guo et al., 2023; Xu et al., 2024; Yan et al., 2024). In Beijing, MRMP detection rates were 74.43%, 90.57%, and 93.94% in 2018, 2019, and 2020, respectively (Wang et al., 2022). In our study, we found that 100% (99/99) of samples carried an A2063G or A2064G transition, with A2063G accounting for 99%. Taken together, these data suggest that China has the highest incidence of MRMP worldwide, with a notable increase in Beijing over the past few years, posing difficulties for treatment.

Epidemic cycles of *M. pneumoniae* may be associated with genotype shifts which occur every 8–10 years (Yan et al., 2019b; Xiao et al., 2020). Before and during the last worldwide pandemic, the P1-1 and 4-5-7-2 genotype strains showed decreasing trends in China, with P1-1 strains decreasing from 76.4% in 2019 to 50% in 2021, and 4-5-7-2 strains decreasing from 84.49% in 2016 to 70.77% in 2019 (Wang et al., 2021; Li et al., 2022). In the current study, the predominant genotypes were P1-1 (76.1%, 54/71) and 4-5-7-2 (73.7%, 73/99), with failure of 28 specimens, probably due to nucleic acid concentrations below that required for the P1-RFLP method for P1 gene typing. These rates were higher than those in previous studies, suggesting a genotype shift phenomenon in late 2023 in Beijing, which may have contributed to the epidemic. Previous studies showed associations between 4-5-7-2 and macrolide resistance, 3-5-6-2 and macrolide susceptibility. P1-2 and 3-5-6-2 indicated that these variants put patients at higher risk for progressing to SMPP (Yan et al., 2015, 2016). However, we found that all *M. pneumoniae*-positive samples harbored macrolide resistance-associated mutations, regardless of genotype, and no significant associations were found between genotypes of *M. pneumoniae* and the severity of MPP. The associations between genotypes and the severity of MPP, genotypes and macrolide resistance-associated mutations require further exploration.

The incidence of SMPP has increased in China in recent years, accounting for 16.0% of MPP cases from 2014 to 2020 in Suzhou, 26.0% in 2021 in Tianjin, and 50.6% in 2022 in Hebei (Lv et al., 2022; Li et al., 2024; Yang et al., 2024). Host cell-mediated immunity is involved in the pathogenesis of *M. pneumoniae* infection (Meyer Sauteur et al., 2023). SMPP patients have more pronounced inflammatory reactions and excessive immune responses, with higher levels of CRP, neutrophils, LDH, ALT, and D-Dimer, longer duration of fever and hospital stay, and lower levels of lymphocytes and CD4(+)T cells, consistent with our findings (Zheng et al., 2021; Li et al., 2024; Yang et al., 2024). However, in our study, the median duration of fever in all patients was 10.0 days (IQR, 8.0–13.0), which is longer than that previously reported in pediatric patients infected with MRMP (8.6–9.8 days; Cheong et al., 2016; Yang et al., 2019). This discrepancy might be attributable to higher levels of macrolide resistance and should be verified by measuring the minimum inhibitory concentration of macrolides against *M. pneumoniae*. Persistent high fever within 72 h after treatment indicates the risk of SMPP (Yan et al., 2024). We found that 100% (40/40) of azithromycin-treated patients were still febrile without significant improvements 72 h after azithromycin administration, suggesting that a high level of macrolide resistance may have been one cause of the SMPP in this epidemic.

The co-infection rate of *M. pneumoniae* and other pathogens ranged from 10 to 56.1%, with the most common co-infected pathogens being *Streptococcus pneumoniae*, *Epstein–Barr virus*, *Human parainfluenza virus*, *Respiratory syncytial virus*, and *Rhinovirus* (Yan et al., 2015; Li et al., 2024; Yang et al., 2024). Co-infection can increase the chance of extrapulmonary complications, aggravate MPP, and even lead to death. In our study, 41.1% (30/73) of MPP patients were co-infected, with *Epstein-Barr virus* (30.0%, 9/30) being the predominant co-infected pathogen. However, we found no associations between co-infection and *M. pneumoniae* genotypes, or between co-infection and SMPP, which might be attributable to our small sample size.

Complications of MPP develop in response to direct effects, such as cytokine release in the inflammation site where *M. pneumoniae* presents, or indirect effect, such as autoimmunity or immune complex production (Waites et al., 2017). The higher incidence of extrapulmonary complications in macrolide-resistant MPP patients compared with that in macrolide-sensitive MPP patients might be linked with stronger host responses induced by more persistent *M. pneumoniae* stimulation. Previous studies showed that incidence rates of macrolide-resistant MPP extrapulmonary complications ranged from 29.6% to 38.0% (Zhou et al., 2014). In our study, the incidence of extrapulmonary complications (54.8%, 40/73) was higher than those in previous reports, suggesting that pneumonia was more severe in this epidemic. In previous reports of MPP in children, predominant complications were digestive disorders, pulmonary consolidation, and pleural effusion (Zhou et al., 2014; Cho et al., 2019; Yan et al., 2019b; Zheng et al., 2021; Yang et al., 2024). Here, we found that the predominant complications were blood system diseases (61.64%, 45/73) and pulmonary consolidation (53.3%, 39/73). Pulmonary consolidation develops because of an excessive cell-mediated immune response of the host, which induces higher interleukin levels and leads to diffuse alveolar damage, resulting in large amounts of fibrous exudates visible in the alveolar cavity. Pulmonary consolidation and pleural effusion are both associated with more severe clinical features, and patients with consolidation have a higher incidence of pleural effusion (Cho et al., 2019). Patients with abnormal coagulation function accounted for 82.2% patients with hematological complications, which was confirmed by the elevated level of D-Dimer, a degradation product of fibrin that is considered to reflect thrombin and fibrinolytic activities. The level of D-Dimer is positively correlated with the severity of MPP (Zheng et al., 2021). Thus, the high incidence of complications associated with severe manifestations in the current cohort suggested that the conditions of patients were more serious than those in previous outbreaks.

This study had several limitations. First, our sample size was small. Second, the specimens were mainly collected from inpatients, whose symptoms were more severe than those in outpatients; thus, the clinical features of the outpatient group require further analysis.

## 5 Conclusion

The *M. pneumoniae* epidemic that occurred in Beijing, China in late 2023 featured 4-5-7-2 and P1-1 as the predominant circulating strains and showed a younger age trend. All of the *M. pneumoniae* isolates in this epidemic carried macrolide-resistance associated mutations. MPP patients in this cohort had longer fever duration, with higher proportions of patients with SMPP disease and complications, compared with previous reports. MRMP and genotype shift may be the causative factors of the 2023 epidemic.

## Data availability statement

The original contributions presented in the study are included in the article/Supplementary material, further inquiries can be directed to the corresponding authors.

## Author contributions

YC: Writing – original draft. XJ: Writing – original draft. YG: Writing – original draft, Methodology, Formal analysis. XR: Writing – original draft. BD: Writing – original draft. HZ: Writing – original draft. YF: Writing – original draft. GX: Writing – original draft. JC: Writing – original draft. LG: Writing – original draft. JF: Writing – original draft. ZF: Writing – original draft. TF: Writing – original draft. ZX: Writing – original draft. ZY: Writing – original draft. YY: Writing – original draft. SZ: Writing – original draft. LH: Writing – original draft. YK: Writing – original draft. LC: Writing – original draft. CY: Writing – original draft, Writing – review & editing. JY: Writing – original draft, Writing – review & editing.
