# Supplementary material for: Increased macrolide resistance rate of Mycoplasma pneumoniae correlated with epidemic in Beijing, China in 2023
Source: Front Microbiol. 2024 Aug 6;15:1449511. doi: 10.3389/fmicb.2024.1449511 (PMC11337199; doi:10.3389/fmicb.2024.1449511)
Supplement: Supplementary file 1 [file Data_Sheet_1.pdf]

Supplementary Material

1 Supplementary Tables

Supplementary Table 1 Laboratory results of SMPP and GMPP patients

	SMPP (n=41)	GMPP (n=32)	*P* value
WBC (×10^9/L)	9.38 (6.70–12.69)	9.07 (6.16–11.86)	0.805
Neutrophil rate	0.65 (0.57–0.77)	0.53 (0.46–0.73)	0.011
Lymphocyte rate	0.26 (0.17–0.35)	0.39 (0.19–0.43)	0.008
Hemoglobin (g/L)	129 (117–135)	133 (124–140)	0.026
Platelet (×10^9/L)	360 (247–483)	352 (227–474)	0.934
C-reactive protein (mg/L)	33.0 (13.0–61.5)	12.7 (10.0–21.1)	0.001
LDH (U/L)	311 (252–440)	232 (205–260)	0.000
Albumin (g/L)	42.0 (37.1–44.2)	43.8 (42.4–45.9)	0.003
ALT (U/L)	17.5 (12.7–30.0)	13.3 (10.4–19.3)	0.022
D-Dimer (mg/LFEU)	1.63 (0.69–4.82)	0.51 (0.27–0.64)	0.000
CD4(+) T cell(/μL)	590 (332–901)	947 (710–1400)	0.001
CD8(+) T cell (/μL)	536 (335–717)	694 (462–882)	0.119
CD4(+)/CD8(+) T cell	1.17 (0.95–1.39)	1.43 (1.16–2.02)	0.005

Supplementary Table 2 Associations between *M. pneumoniae* genotypes and the severity of MPP

	SMPP (n=41)	GMPP (n=32)	*P* value
**MLVA type**			0.495
M4-5-7-2	50.9% (27/53)	49.1% (26/53)	
M3-5-6-2	66.7% (10/15)	33.3% (5/15)	
M5-5-7-2	100.0% (1/1)	-	
M3-6-6-2	100.0% (1/1)	-	

M4-5-7-3	100.0% (1/1)	-	
M4-4-7-2	100.0% (1/1)	-	
M3-5-7-2	-	100.0% (1/1)	
**P1 subtype**			0.338
P1-1	50.0% (18/36)	50.0% (18/36)	
P1-2	66.7% (2/3)	33.3% (1/3)	
P1-2c	77.8% (7/9)	22.2% (2/9)	

Supplementary Table 3 Patient information of co-infection and MP-mono infection, and associations with *M. pneumoniae* genotypes

	Co-infection(n=30)	MP-mono infection(n=43)	*P* value
**Age**	7.0 (5.7–9.5)	8.7(6.7–10.8)	0.026
**Sex** (male/female)	1.0 (15/15)	0.9 (20/23)	0.769
**MLVA type**			0.764
M4-5-7-2	44.4% (24/54)	55.6% (30/54)	
M3-5-6-2	33.3% (5/15)	66.7% (10/15)	
M5-5-7-2	-	100% (1/1)	
M3-6-6-2	-	100% (1/1)	
M3-5-7-2	-	100% (1/1)	
M4-4-7-2	100% (1/1)	-	
**P1 genotype**			0.126
P1-1	38.9% (14/36)	61.1% (22/36)	
P1-2	75.0% (3/4)	25.0% (1/4)	
P1-2c	18.2% (2/11)	81.8% (9/11)	

Supplementary Table 4 Predominant complications of MPP patients with mono-*M. pneumoniae* infection and associations between complications and *M. pneumoniae* genotypes

Complications	NO. of patient (n=42)	P1-1 (n=21)	P1-2 (n=1)	P1-2c (n=9)	*P* value	M4-5-7-2 (n=28)	M3-5-6-2 (n=10)	M3-5-7-2 (n=1)	M3-6-6-2 (n=1)	M4-5-7-3 (n=1)	M5-5-7-2 (n=1)	*P* value
Pulmonary consolidation	57.1% (24/42)	57.1% (12/21)	100.0% (1/1)	44.4% (4/9)	0.832	57.1% (16/28)	40.0% (4/10)	100% (1/1)	100% (1/1)	100% (1/1)	100% (1/1)	0.774
Blood system diseases	59.5% (25/42)	47.6% (10/21)	100.0% (1/1)	88.9% (8/9)	0.069	53.6% (15/28)	60.0% (6/10)	100% (1/1)	100% (1/1)	100% (1/1)	100% (1/1)	1.000
